# Analysis of MicroRNA Signature Differentially Expressed in Pancreatic Islet Cells Treated with Pancreatic Cancer-Derived Exosomes

**DOI:** 10.3390/ijms241814301

**Published:** 2023-09-19

**Authors:** Young-gon Kim, Jisook Park, Eun Young Park, Sang-Mi Kim, Soo-Youn Lee

**Affiliations:** 1 Department of Laboratory Medicine and Genetics, Samsung Medical Center, Sungkyunkwan University School of Medicine, Seoul 06351, Republic of Korea; younggonn.kim@samsung.com (Y.-g.K.); sangmi22.kim@samsung.com (S.-M.K.); 2 Samsung Biomedical Research Institute, Samsung Medical Center, Seoul 06351, Republic of Korea; js944837@hanmail.net (J.P.); noringi@naver.com (E.Y.P.); 3 Department of Clinical Pharmacology and Therapeutics, Samsung Medical Center, Seoul 06351, Republic of Korea

**Keywords:** pancreatic cancer, diabetes mellitus, miRNA, exosome, insulin resistance

## Abstract

Since the majority of patients with pancreatic cancer (PC) develop insulin resistance and/or diabetes mellitus (DM) prior to PC diagnosis, PC-induced diabetes mellitus (PC-DM) has been a focus for a potential platform for PC detection. In previous studies, the PC-derived exosomes were shown to contain the mediators of PC-DM. In the present study, the response of normal pancreatic islet cells to the PC-derived exosomes was investigated to determine the potential biomarkers for PC-DM, and consequently, for PC. Specifically, changes in microRNA (miRNA) expression were evaluated. The miRNA specimens were prepared from the untreated islet cells as well as the islet cells treated with the PC-derived exosomes (from 50 patients) and the healthy-derived exosomes (from 50 individuals). The specimens were subjected to next-generation sequencing and bioinformatic analysis to determine the differentially expressed miRNAs (DEmiRNAs) only in the specimens treated with the PC-derived exosomes. Consequently, 24 candidate miRNA markers, including IRS1-modulating miRNAs such as hsa-miR-144-5p, hsa-miR-3148, and hsa-miR-3133, were proposed. The proposed miRNAs showed relevance to DM and/or insulin resistance in a literature review and pathway analysis, indicating a potential association with PC-DM. Due to the novel approach used in this study, additional evidence from future studies could corroborate the value of the miRNA markers discovered.

## 1. Introduction

Pancreatic cancer (PC) is a highly fatal disease with a well-known dismal prognosis. The 5-year survival rate at the time of diagnosis is lower than 10% because 80–85% of patients present with an unresectable disease [1,2]. Despite the evident causality between late diagnosis and poor prognosis, advances in diagnostics have been slow, rendering PC an increasingly common cause of cancer-related mortality.

Since the signs and symptoms of PC are vague and usually manifest at late stages, the role of diagnostic modalities is essential for early detection. However, imaging-based screening is recommended only for high-risk individuals because of suboptimal sensitivity and specificity [2]. Liquid biopsy-based biomarkers, such as CA19-9, HbA1C, exosomes, and circulating-tumor DNA (ctDNA), have been investigated, but none have yet been approved as a standalone biomarker [2,3,4]. Although ctDNA assays are mostly being studied for use in the early detection of multiple cancers, exosomes have also been investigated for a potential role in the early diagnosis of cancer, especially as adjunctive markers with synergistic information [4,5,6].

Exosomes are the predominant extracellular vesicles secreted by various types of cells including cancer cells. Exosomes have been shown to play a key role in intercellular communication by transporting messenger RNA (mRNA), microRNA (miRNA), and proteins [7,8]. Exosomes have been shown to have a significant influence on tumor initiation, growth, progression, metastasis, and drug resistance in various types of cancers including PC [9,10,11,12,13,14]. Although approved exosome biomarkers for cancer diagnosis do not exist to date, numerous markers reportedly have the potential for cancer diagnosis [15]. In particular, miRNA has attracted increased attention due to its abundance in exosomes and regulatory functions [6,15]. Specifically, miR-191, miR-21, and miR-451a have been suggested as exosomal biomarkers for PC diagnosis [16].

Based on the observation that patients with newly diagnosed PC frequently have recently diagnosed diabetes mellitus (DM), DM has been consistently suggested as a potential platform for early PC detection [17,18,19,20,21,22]. While DM is an established risk factor for PC, PC has also been increasingly recognized as a cause of DM [19,20,21,22]. Up to 85% of patients with PC have DM or hyperglycemia, which frequently manifests as early as 2–3 years before a diagnosis of PC [22]. Therefore, it is reasonable to assume that the discovery of a biomarker for PC-induced DM (PC-DM) could contribute to the early diagnosis of PC. Although some markers can be used for distinction between type 1 DM and type 2 DM, such as the serum c-peptide level [23], a marker has yet to be discovered for the differential diagnosis of PC-DM.

A potential role of the PC-derived exosomes in the development of PC-DM has been investigated. Adrenomedullin, a protein marker in the PC-exosomes, was designated as a candidate mediator of β-cell dysfunction in PC [18,24]. Among miRNA markers derived from the PC exosomes, miR-6796-3p, miR-6763-5p, miR-4750-3p, and miR-197-3p were reported to disrupt glucose homeostasis by downregulating the glucose-dependent insulinotropic peptide (GIP) and the glucagon-like peptide-1 (GLP-1) [25]. In another study, miR-19a was reported to cause decreased insulin secretion by targeting Neurod1 [26]. In a study in which the insulin resistance caused by PC was investigated, PC-derived miRNAs, such as miR-666-3p, miR-540-3p, miR-125b-5p, miR-450b-3p, miR-883b-5p, 666-3p, miR-450b-3p, and miR-151-3p, were found to be capable of inducing insulin resistance in the skeletal muscle cells through the PI3K/Akt/FoxO1 signaling pathways [27]. In those studies, miRNAs were directly isolated from the PC-derived exosomes [16,25,27]. To the best of our knowledge, miRNAs produced from the normal islet cells in response to the PC-derived exosomes have not been investigated.

**Figure 1:**
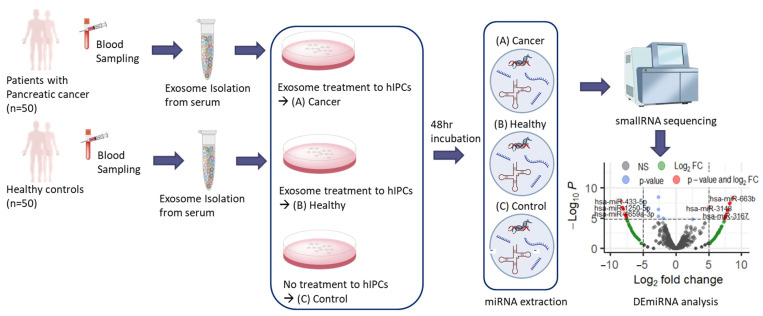
Outline of experimental design. The miRNA specimens, Cancer, Healthy, and Control were prepared and DEmiRNA analysis was performed to determine miRNA markers differentially expressed from (A) Cancer. Acronyms: miRNA, microRNA; hIPCs, human pancreatic islet-derived precursor cells; DEmiRNA, differentially expressed miRNA.

Based on the widespread assumption that the PC-derived exosomes can induce PC-DM, the changes in miRNA expression in the islet cells during the exosome-induced PC-DM development was investigated in the present study. To identify potential miRNA biomarkers for PC-DM, and consequently, for PC, the miRNA expression from the normal islet cells in response to the PC-derived exosomes was analyzed (Figure 1).

## 2. Results

### 2.1. DEmiRNAs

**Figure 2:**
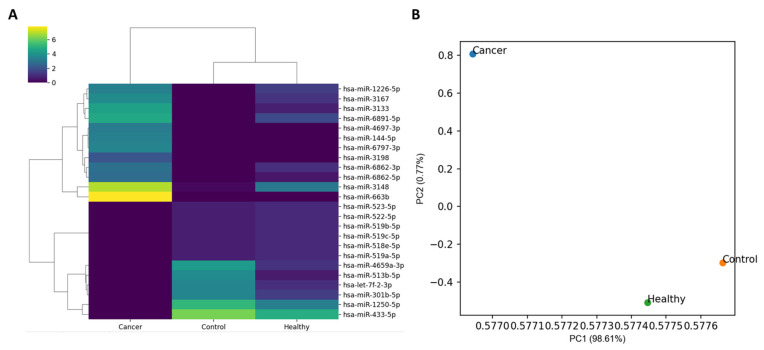
Clustering pattern of normalized expression in three specimens: Cancer, Healthy, and Control. (**A**) Control and Healthy showed clustering based on the unsupervised clustering analysis. For visualization, 24 miRNA markers finally chosen in this study were used for heatmap construction. (**B**) In the two-dimensional scatter plot produced based on PCA analysis, Cancer and Healthy were located close and Cancer was distant from both Healthy and Control. In this analysis, all 2656 mature miRNA markers were used.

The number of miRNA markers used in the expression analysis was 2656. The heatmap of the normalized expression of miRNA markers from three specimens and the scatter plot drawn from the principal component analysis (PCA) are shown in Figure 2. Unsupervised clustering analysis revealed the clustering of Control and Healthy. Cancer was distant from both Healthy and Control in the scatter plot and Control and Healthy were located relatively closer.

**Figure 3:**
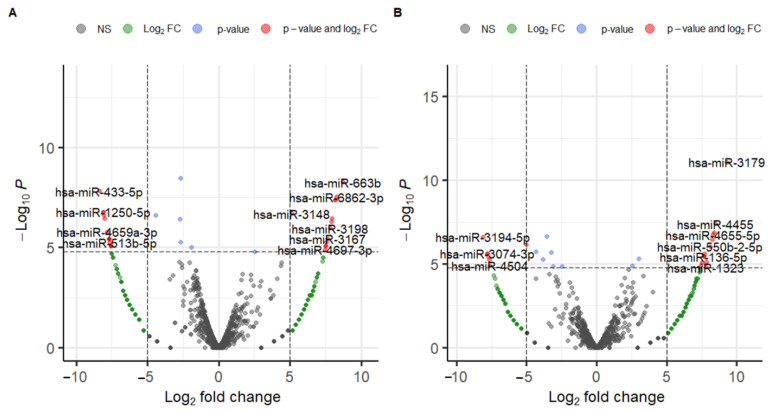
Volcano plots produced from DEmiRNA analysis. (**A**) DEmiRNA1 (Cancer vs. Control), and (**B**) DEmiRNA2 (Healthy vs. Control).

The results of the DEmiRNA analysis, DEmiRNA1, and DEmiRNA2 are shown in Figure 3 and Tables S1 and S2. DEmiRNA1 (Cancer vs. Control) revealed 13 upregulated miRNAs and 16 downregulated miRNAs (Table S1). DEmiRNA2 (Healthy vs. Control) revealed 14 upregulated markers and seven downregulated markers (Table S2). Among 13 upregulated miRNAs in DEmiRNA1, one was also upregulated in DEmiRNA2, and among 16 downregulated miRNAs in DEmiRNA1, four were also downregulated in DEmiRNA2. Therefore, the candidate markers derived from the DEmiRNA analysis numbered 24, consisting of 12 upregulated miRNAs and 12 downregulated miRNAs (Table 1 and Table S3).

The exact same log_2_FC, log_2_CPM, and p-values were observed in six downregulated miRNAs: hsa-miR-518e-5p, hsa-miR-519a-5p, hsa-miR-519b-5p, hsa-miR-519c-5p, hsa-miR-522-5p, and hsa-miR-523-5p. These miRNAs were defined as similar miRNAs in miRDB sharing the same seed sequence of CUCUAGAGGGAAGCGCUUUCUG [28]. Based on the exact same expression levels of these miRNAs, we reasoned that this redundancy was caused by sequence ambiguity during the bioinformatic process. Therefore, these miRNAs were collapsed in the downstream analysis into one miRNA, hsa-miR-518e-5p, resulting in 19 candidate miRNA markers. The number of genes in the union of target genes of the 19 miRNAs was 3206 (Table S3).

**Figure 4:**
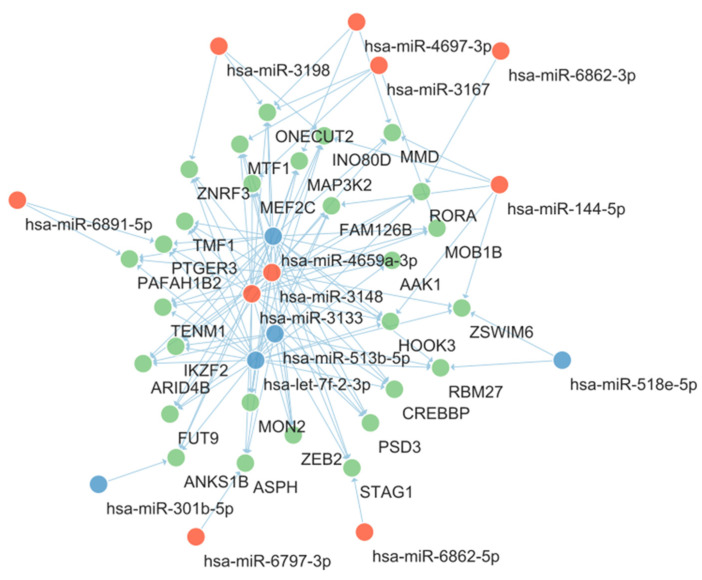
miRNA-gene network showing the interaction between candidate miRNAs and predicted target genes. The red nodes represent upregulated miRNAs and the blue nodes represent downregulated miRNAs. The green nodes represent predicted target genes. Acronyms: miRNA, microRNA.

To visualize the interactions between the candidate miRNA markers and genes, a miRNA-gene network was constructed. To reduce the complexities in the network, among the 3206 predicted target genes, 28 were targeted by at least four candidate miRNAs and were included in the network construction. The resultant miRNA-gene network is shown in Figure 4. The nodes were arranged automatically with Cytoscape software (version 3.9.1) according to the Prefuse Force Directed Layout. Among candidate miRNAs, hsa-miR-4659a-3p, hsa-miR-3148, hsa-miR-3133, hsa-let-7f-2-3p, and hsa-miR-513b-5p were located at the central portion of the network and their degrees were higher than other miRNAs (Table 1).

Among the genes included in Figure 4, TMF1 and PTGER3 were previously reported to be involved in glucose and insulin homeostasis. TMF1 was reported to be involved in the insulin-directed, GLUT4-mediated glucose uptake [29]. In that study, the absence of TMF1 led to the retention of GLUT4 in perinuclear compartments and hyperglycemia was induced in TMF1 knockout mice. In the miRNA-gene network in Figure 4, TMF1 had interactions with four centrally located miRNA markers as described above, which are hsa-miR-4659a-3p, hsa-miR-3148, hsa-miR-3133, and hsa-let-7f-2-3p. PTGER3 is the gene for prostaglandin E receptor 3 (EP3), which is reported to be upregulated in diabetes patients [30]. The activation of EP3 results in a decrease in intracellular cAMP, a blunting glucose-stimulated insulin secretion. PTGER3 showed interaction with three centrally located miRNA markers: miR-4659a-3p, hsa-miR-3133, and hsa-let-7f-2-3p.

### 2.2. KEGG Pathway Enrichment Analysis

**Figure 5:**
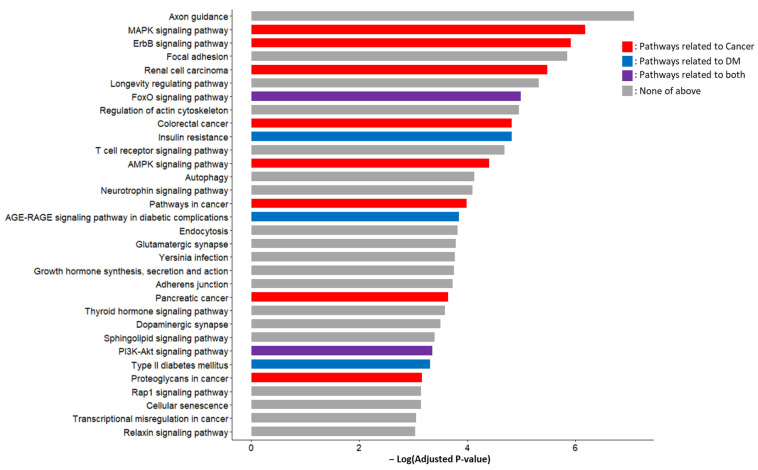
KEGG pathways enriched by predicted target genes of candidate miRNA markers. In addition to the cancer-related pathways such as pathways in cancer, PC, and various signaling pathways, DM-related pathways such as insulin resistance, type 2 DM, PI3K-Akt signaling pathway, FoxO signaling pathway, and AGE-RAGE signaling pathways in diabetic complications showed higher significance. Acronyms: KEGG, Kyoto Encyclopedia of Genes and Genomes; miRNA, microRNA; PC, pancreatic cancer; DM, diabetes mellitus.

The unions of the predicted target genes of 19 candidate miRNA markers (3206 genes) were used as the input for the KEGG pathway and GO function analyses. The KEGG pathway analysis results included the various pathways associated with cancer and/or DM (Figure 5). Among the 32 pathways with an adjusted *p*-value < 0.01, five were DM-related, including two of them that were related to both DM and cancer. The DM-related pathways included type 2 diabetes mellitus, insulin resistance, the AGE-RAGE signaling pathways in diabetic complications, the FoxO signaling pathway, and the PI3K-Akt signaling pathway. Notably, the FoxO signaling pathway and the PI3K-Akt signaling pathway were reported in a previous study to mediate the insulin resistance caused by PC [27].

**Figure 6:**
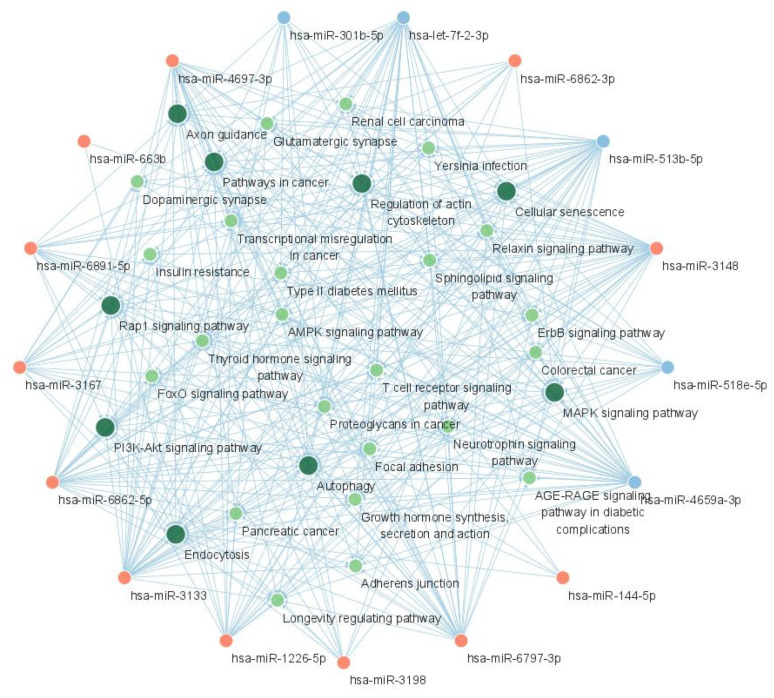
The miRNA-KEGG network showing the interaction between candidate miRNAs and KEGG pathways. The red nodes represent upregulated miRNAs and the blue nodes represent downregulated miRNAs. The green nodes represent KEGG pathways and larger green nodes represent KEGG pathways with degree >17. Acronyms: miRNA, microRNA; KEGG, Kyoto Encyclopedia of Genes and Genomes.

The miRNA-KEGG network was constructed using the chain relationship of the miRNA-gene and gene-KEGG. The resultant network is shown in Figure 6. In addition to the PI3K-Akt signaling pathway, the MAPK signaling pathway, the pathways in cancer, and the Rap1 signaling pathway showed central interactions in this network.

### 2.3. GO Function Enrichment Analysis

**Figure 7:**
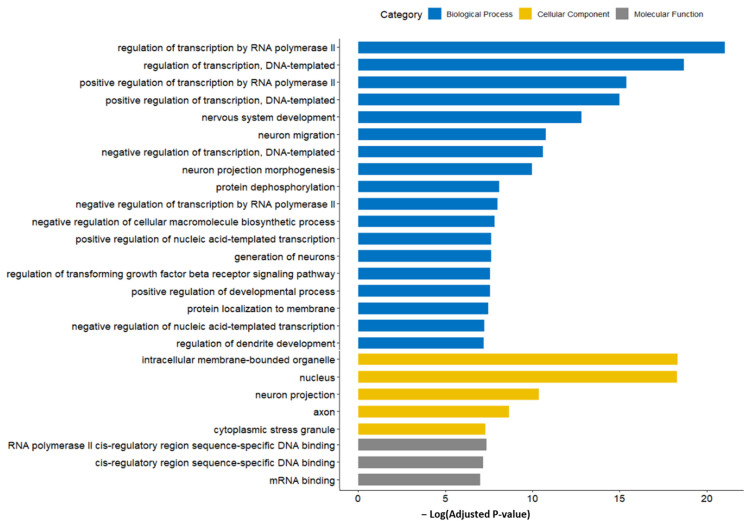
GO terms enriched by predicted target genes of candidate miRNA markers. Terms related to translation regulation showed higher significance. Acronyms: GO, gene ontology; miRNA, microRNA.

**Figure 8:**
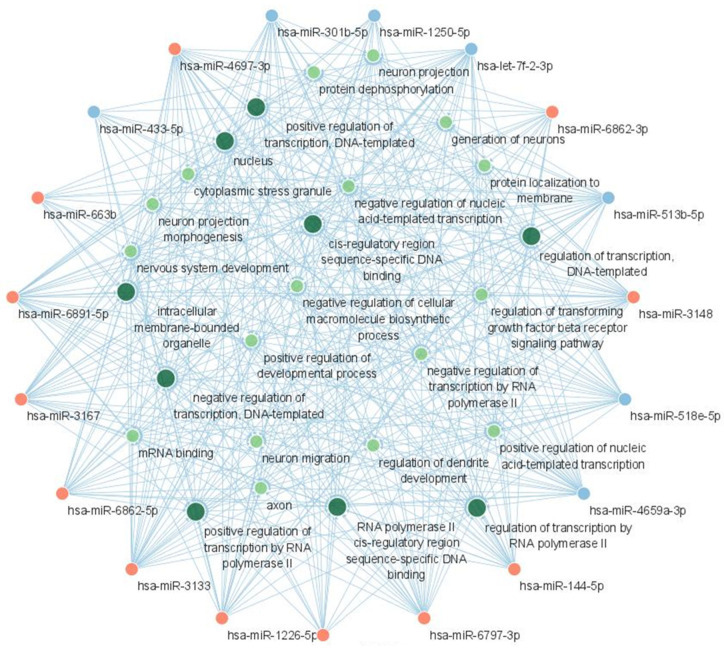
miRNA-GO network showing the interactions between candidate miRNAs and GO terms. The red nodes represent upregulated miRNAs and the blue nodes represent downregulated miRNAs. The green nodes represent GO terms and larger green nodes represent GO terms with degree > 12. Acronyms: miRNA, microRNA; GO, gene ontology.

The result of GO function analysis is shown in Figure 7. The GO terms with the highest significance (lowest adjusted *p*-value) were mostly associated with the translation regulation, including the biological processes of the regulation of transcription by RNA polymerase II; the regulation of transcription, the DNA-templated; positive regulation of transcription by RNA polymerase II; and the positive regulation of transcription, DNA-templated. Among cellular components, the intracellular membrane-bounded organelles and nucleus showed the highest significance. The miRNA-GO network was constructed in a similar manner to the miRNA-KEGG network (Figure 8). Again, the terms associated with translation regulation (including all terms listed above) showed a central interaction in this network.

### 2.4. Expression Pattern of Candidate miRNA Markers in Non-Treated Cancer Cell Lines

To estimate the baseline expression of 24 candidate miRNA markers in the PC cells, the expression patterns of candidate markers in commercial PC cell lines were measured without exosome treatment. Two samples were prepared using Capan-2 and MIA PaCa-2 cell lines and DEmiRNA analyses were performed as described in the Materials and Methods section. The results of DEmiRNA3 (Capan-2 versus hIPC Control) and DEmiRNA4 (MIA PaCa-2 versus hIPC Control) are described in Table S4. In DEmiRNA3, seven markers (hsa-miR-1250-5p, hsa-miR-433-5p, hsa-miR-519a-5p, hsa-miR-519b-5p, hsa-miR-519c-5p, hsa-miR-522-5p, and hsa-miR-523-5p) were statistically significant after the Bonferroni correction and all of them showed the same expression tendency as DEmiRNA1, which is the main result of this study. In DEmiRNA4, 11 markers (hsa-let-7f-2-3p, hsa-miR-1250-5p, hsa-miR-301b-5p, hsa-miR-433-5p, hsa-miR-513b-5p, hsa-miR-518e-5p, hsa-miR-519a-5p, hsa-miR-519b-5p, hsa-miR-519c-5p, hsa-miR-522-5p, and hsa-miR-523-5p) were statistically significant and 10 (90.9%) showed the same tendency as DEmiRNA1.

## 3. Discussion

The potential role of the PC-derived exosomes in the development of PC-DM has been widely accepted [18,24,25,26,27,31,32]. Many studies were performed to determine the exosomal mediator of PC-DM, and the protein adrenomedullin has been proposed with the highest level of evidence to date [18,32]. However, the response of the pancreatic islet cells to the PC-derived exosomes has not been investigated. In the present study, the changes in miRNA expression were investigated among the various potential responses of the islet cells to the PC-derived exosomes.

First, whether the PC-derived exosomes can influence the miRNA expression of the islet cells was tested using the basic expression analysis. In addition, the effect size of the PC-derived exosomes was compared with the healthy-derived exosomes. In the clustering analysis of the normalized miRNA expression including PCA analysis, the miRNA expression pattern of Healthy was close to Control, and that of Cancer was distant from those of both Healthy and Control. This result confirmed the influence of the PC-derived exosomes on miRNA expression, which was stronger than the healthy-derived exosomes.

Subsequently, DEmiRNA analysis was designed to measure the expression changes caused only by the PC-derived exosomes and not the healthy-derived exosomes. It was found that 19 (24, including redundancies) miRNAs were differentially expressed only in the islet cells treated with the PC-derived exosomes. Among them, hsa-miR-144-5p has been repeatedly reported for its relevance to insulin resistance and type 2 DM [33,34,35,36,37,38]. In patients with type 2 DM, hsa-miR-144-5p was reported to impair insulin signaling by inhibiting the expression of IRS1 [33]. The hsa-miR-144 blood level was reported to inversely correlate with the blood insulin level and to correlate with the serum glucose and HbA1C level [34,38]. These findings are consistent with the result of this study in which hsa-miR-144-5p was upregulated in Cancer. Similarly, hsa-miR-3148 and hsa-miR-3133, which were upregulated in Cancer and located in the central portion of the miRNA-gene network (Figure 4), were also predicted to target IRS1 (Table S3). In particular, hsa-miR-3148 was reported to impair the insulin signaling pathway by downregulating IRS1, causing insulin resistance [39]. IRS1 and the related miRNAs, hsa-miR-144-5p, hsa-miR-3148, and hsa-miR-3133, might play an important role in the development of PC-DM.

The additional miRNA markers showed a consistent expression pattern with those reported in the literature. The hsa-miR-4697-3p level, which was upregulated in Cancer in the present study, was reported to show a significant correlation with the blood glucose level and was upregulated in metabolically unhealthy subjects [40]. Hsa-miR-518e-5p, which was downregulated in Cancer in the present study, was reportedly downregulated in patients with metabolic syndrome [41]. In addition, patients who were successfully treated for DM showed the opposite trend in miRNA expression. Patients who responded to dipeptidyl peptidase-IV treatment showed downregulated hsa-miR-1226-5p, which was upregulated in Cancer in the present study [42].

There are five DM-related pathways under the category of Endocrine and metabolic disease in the entire list of the KEGG pathways (https://www.genome.jp/kegg/pathway.html#disease, accessed 10 August 2023), which include type 2 diabetes mellitus, type 1 diabetes mellitus, maturity onset diabetes of the young, insulin resistance, and the AGE-RAGE signaling pathway in diabetic complications. Considering the acquired nature of PC-DM and its relevance to insulin resistance, type 1 diabetes mellitus and maturity-onset diabetes of the young likely have minimal relevance to PC-DM. The three remaining pathways were retrieved via the miRNAs discovered in the present study, supporting their potential value as PC-DM markers. In addition, the observed relevance to the FoxO signaling pathway and the PI3K-Akt signaling pathway, which were reported to mediate the insulin resistance caused by PC [27], supports the potential role of the proposed markers in the PC-DM. IRS1, which was proposed as a key player in the PC-DM development above, acts via the PI3K/Akt/FoxO pathway, indicating the importance of both IRS1 and these pathways [43,44].

The present study had several limitations. First, the miRNA samples could not be prepared with biological replicates. Instead, to reduce the effect of random variation, a higher threshold of 5 for the log_2_ fold change was used. Conversely, this might have resulted in missing significant miRNA markers. Second, the pancreatic islet cells used in this study were not mature β-cells. However, as mentioned in the Methods section, hIPCs are human-derived progenitors of the β-cells that are widely used in human DM research [45,46,47,48]. Future studies including similar experiments with mature β-cells could provide additional insights regarding PC-DM. Thirdly, not all PC patients included had PC-DM (40% of patients had DM at the time of enrollment, as described in the Materials and Methods section). Constituting the Cancer group purely with patients with DM or insulin resistance could have resulted in a more rigorous study design to find out the cause of insulin resistance in PC patients. However, the role of PC patients in this study was as exosome donors rather than as the subjects of the measurement. Assuming that the PC-DM development is determined by the combination of factors, including host factors and external stimuli such as the cancer-derived exosomes, the exosomes derived from PC patients without DM still might contain diabetogenic components. We believe that the pooled exosomes derived from 50 PC patients, of which 40% were diagnosed with DM, should have been effective in the derivation of diabetogenic changes, if existing.

In conclusion, 24 miRNAs were differentially expressed in the pancreatic islet cells treated with the PC-derived exosomes. Based on the relationship of these miRNAs to DM determined from the literature review and pathway analysis, we propose these miRNAs as potential biomarkers for PC-DM. Due to the novelty of the experimental design, additional evidence from future studies could strengthen the value of these markers.

## 4. Materials and Methods

The general study concept is depicted in Figure 1. Three miRNA samples, Cancer, Healthy, and Control, were prepared and analyzed using the procedures described below.

### 4.1. Participants

The Institutional Review Board (IRB) of Samsung Medical Center (SMC), Seoul, Republic of Korea (IRB No.: SMC 2021-02-153-001) approved this study. To prepare the Cancer specimen, leftover serum specimens from CA 19-9 tests were collected from 50 patients with PC (20 patients diagnosed with diabetes mellitus, 21 stage I/II, and 29 stage III/IV). For the Healthy specimen, leftover serum specimens from routine chemistry tests were collected from 50 individuals without a previous history of cancer.

### 4.2. Exosome Isolation

From each patient, 100 μL of serum was used for the exosome isolation. The multiple-cycle polymeric extracellular vesicle (EV) precipitation method, designed for high purity EV isolation in a previous study [49], was used. The ExoQuick exosome precipitation solution (System Biosciences, Palo Alto, CA, USA) was used as previously described [49].

### 4.3. Culture and Exosome Treatment of Human Pancreatic Islet-Derived Precursor Cells (hIPCs)

Immortalized human pancreatic islet-derived precursor cells (hIPCs) were purchased from ABM (Vancouver, BC, Canada). The hIPCs are a type of mesenchymal stem/stromal cell (MSCs) capable of proliferating and differentiating into the hormone-expressing islet cells such as β-cells [45]. Since mature β-cells do not easily proliferate in the culture and most artificial insulin-secreting cell lines are not human-derived, hIPCs are widely used in human DM research [45,46,47,48]. Three cell lines of hIPCs were incubated at 37 °C and 5% CO_2_ in the Prigrow II medium with 10% fetal bovine serum (FBS), to the density of 1 × 10^6^ cells/cm^2^. Subsequently, one cell line (Cancer) was treated with the pooled exosomes from PC patients, a second cell line (Healthy) was treated with the pooled exosomes from the healthy controls, and the third cell line (Control) was not treated. The amount of the pooled exosomes treated to Cancer and Healthy were adjusted so that the final protein concentration in the culture media fits 50 µg/mL using the Pierce^TM^ BCA Protein Assay Kit (Thermo Scientific^TM^, Waltham, MA, USA).

### 4.4. miRNA Extraction

After 48 h of incubation, trypsinization was applied to detach the adherent cells and then the cells were pelleted at low speed (300× *g*, 3 min). The culture media was removed, and total RNA extraction was performed using the RNeasy Mini kit (Qiagen, Redwood City, CA, USA) according to the manufacturer’s instructions. RNA quantity and quality were measured using a NanoDrop spectrophotometer (Nanodrop Technologies, Wilmington, DE, USA) and the Agilent 2100 Bioanalyzer (Agilent Technologies, Inc., Santa Clara, CA, USA).

### 4.5. miRNA Sequencing and Bioinformatic Process

Next generation sequencing [50] was used in the measurement of miRNA expression. The library preparation, miRNA sequencing, and the bioinformatic process (except for running edgeR) were outsourced and performed by Theragen Bio. (Seongnam, Republic of Korea). The libraries were prepared using the NEXTflex Small RNA-Seq Kit V3 (PerkinElmer Inc., Shelton, CT, USA) according to the manufacturer’s instructions. After the adapter ligation and double-stranded cDNA synthesis, fragments approximately 150 bp in size were extracted for sequencing via size selection using gel electrophoresis. Quality of the cDNA libraries was evaluated with the TapeStation 4200 (Agilent Technologies, Inc.), followed by quantification with the KAPA library quantification kit (Kapa Biosystems, Wilmington, MA, USA) according to the manufacturer’s protocol. Sequencing was performed as single-end (50 bp) using Illumina NovaSeq6000 (Illumina Inc., San Diego, CA, USA). Filtered reads were mapped to the reference genome using the aligner Bowtie (version 1.3.0) [51]. The miRNA expression level was measured with mirdeep2 (version 2.0.1.3) [52] using the gene annotation database along with hairpin and mature miRNA sequence information from miRBase [53]. All parameters were set to the default values.

### 4.6. Differentially Expressed miRNA (DEmiRNA) Analysis

Differentially expressed miRNAs (DEmiRNAs) were identified using the R package edgeR [54]. DEmiRNA1 was defined as the DEmiRNA analysis between Cancer versus Control and DEmiRNA2 was defined as Healthy versus Control. DEmiRNA analysis between Cancer versus Healthy was not performed because, in this setting, the markers that show changes only in Healthy and not in Cancer could be considered significant. Instead, to remove the markers that were significant in both Healthy and Cancer, the final candidate markers were chosen by subtracting the resultant markers in DEmiRNA2 from the resultant markers in DEmiRNA1. Bonferroni correction was applied for the correction of errors caused by the multiple tests, and the resultant cutoff for the *p*-value was 0.0000188 (0.05/2656). The cutoff for the log_2_ fold change (FC) was 5.

### 4.7. Pathway and Gene Ontology (GO) Analysis

Potential target genes of the candidate miRNAs were predicted with the miRDB online database [28] (accessed on 19 March 2023) using a threshold target score of 80. Predicted target genes subsequently underwent a gene ontology (GO) analysis for functional analysis and a Kyoto Encyclopedia of Genes and Genomes (KEGG) database analysis to identify the enriched functions and pathways that might be involved. Enrichr (https://maayanlab.cloud/Enrichr/, accessed on 21 March 2023) was used for the GO and KEGG database search. The adjusted p-values provided by Enrichr were used. The KEGG pathways with adjusted *p*-values < 0.01 (instead of 0.05) were utilized in the downstream analysis due to the complexities in the networks caused by the excessive number of nodes. For the similar reason, an adjusted *p*-value cutoff of 10^−5^ was used in the GO function analysis.

To visualize the associations between the candidate miRNA markers and their target genes and pathways, three types of networks were constructed: miRNA versus genes, miRNA versus GO terms, and miRNA versus KEGG pathways. Cytoscape software (version 3.9.1) was used for the network construction. In each network, the output degree was used to measure the effect size of the miRNAs or genes.

### 4.8. Non-Treated Cancer Cell Lines Analysis

Two cancer cell lines, Capan-2 and MIA PaCa-2, were purchased from the Korean Cell Line Bank (Seoul, Republic of Korea). Using these two cell lines, incubation, miRNA extraction, miRNA sequencing, and the bioinformatic process were performed in the same way as described above. Exosomes were not treated to these cell lines. DEmiRNA3 was defined as the DEmiRNA analysis between Capan-2 versus Control (the one prepared using hIPCs), and DEmiRNA4 was defined as MIA PaCa-2 versus Control.

## Figures and Tables

**Table 1:** Candidate miRNA markers derived from DEmiRNA analysis.

miRNA	log_2_FC	log_2_CPM	*p*-Value	Upregulated or Downregulated	Degree in miRNA-Gene Network
hsa-let-7f-2-3p	−7.57	0.93	0.0000083	Downregulated	17
hsa-miR-1226-5p	7.50	1.10	0.0000083	Upregulated	0
hsa-miR-1250-5p	−8.11	1.42	0.0000002	Downregulated	0
hsa-miR-144-5p	7.50	1.10	0.0000083	Upregulated	5
hsa-miR-301b-5p	−7.57	0.93	0.0000083	Downregulated	1
hsa-miR-3133	7.89	1.43	0.0000005	Upregulated	20
hsa-miR-3148	5.14	2.02	0.0000002	Upregulated	25
hsa-miR-3167	7.66	1.24	0.0000036	Upregulated	4
hsa-miR-3198	7.80	1.36	0.0000011	Upregulated	3
hsa-miR-433-5p	−8.38	1.67	<0.0000001	Downregulated	0
hsa-miR-4659a-3p	−7.83	1.17	0.0000016	Downregulated	22
hsa-miR-4697-3p	7.44	1.06	0.0000127	Upregulated	3
hsa-miR-513b-5p	−7.64	1.00	0.0000054	Downregulated	10
hsa-miR-518e-5p	−7.71	1.06	0.0000036	Downregulated	2
hsa-miR-519a-5p	−7.71	1.06	0.0000036	Downregulated	NA
hsa-miR-519b-5p	−7.71	1.06	0.0000036	Downregulated	NA
hsa-miR-519c-5p	−7.71	1.06	0.0000036	Downregulated	NA
hsa-miR-522-5p	−7.71	1.06	0.0000036	Downregulated	NA
hsa-miR-523-5p	−7.71	1.06	0.0000036	Downregulated	NA
hsa-miR-663b	8.70	2.16	<0.0000001	Upregulated	0
hsa-miR-6797-3p	7.55	1.15	0.0000054	Upregulated	1
hsa-miR-6862-3p	8.26	1.76	<0.0000001	Upregulated	1
hsa-miR-6862-5p	8.19	1.70	<0.0000001	Upregulated	1
hsa-miR-6891-5p	7.97	1.50	0.0000003	Upregulated	2

Acronyms: miRNA, microRNA; DEmiRNA, differentially expressed microRNA; FC, fold change; CPM, counts per million; NA, not applicable.

## Data Availability

The data presented in this study are available in the article or Supplementary Materials.

## Supplementary Materials

The supporting information can be downloaded at: https://www.mdpi.com/article/10.3390/ijms241814301/s1.
